# Incidence of tuberculosis among school-going adolescents in South India

**DOI:** 10.1186/s12889-016-3342-0

**Published:** 2016-07-26

**Authors:** Dharma Rao Uppada, Sumithra Selvam, Nelson Jesuraj, Esther L. Lau, T. Mark Doherty, Harleen M. S. Grewal, Mario Vaz, Bernt Lindtjørn, TB Trials Study Group, T. M. Doherty, G D’Souza, H. M. S. Grewal, A. C. Hesseling, A. Jacob, F. Jahnsen, A. V. Kurpad, E. Lau, B. Lindtjorn, R. Macaden, M. Vaz

**Affiliations:** 1 Department of Pharmacology, Rangaraya Medical College, Kakinada, Andhra Pradesh 533001 India; 2 St. John’s Research Institute, Bangalore, India; 3 Formerly at St. John’s Research Institute, Bangalore, India; 4 Aeras, Metro Area, Washington, DC USA; 5 GSK Vaccines, Wavre, Belgium; 6 Department of Clinical Science, Infection, Faculty of Medicine and Dentistry, University of Bergen, Bergen, Norway; 7 Department of Microbiology, Haukeland University Hospital, N-5021 Bergen, Norway; 8 Physiology and Head of Health and Humanities, St. John’s Medical College, Bangalore, India; 9 Centre for International Health, University of Bergen, Bergen, Norway

**Keywords:** Pulmonary tuberculosis, TB incidence, Adolescents, South India, TB vaccine trials

## Abstract

**Background:**

Tuberculosis (TB) incidence data in vaccine target populations, particularly adolescents, are important for designing and powering vaccine clinical trials. Little is known about the incidence of tuberculosis among adolescents in India. The objective of current study is to estimate the incidence of pulmonary tuberculosis (PTB) disease among adolescents attending school in South India using two different surveillance methods (active and passive) and to compare the incidence between the two groups.

**Methods:**

The study was a prospective cohort study with a 2-year follow-up period. The study was conducted in Palamaner, Chittoor District of Andhra Pradesh, South India from February 2007 to July 2010. A random sampling procedure was used to select a subset of schools to enable approximately 8000 subjects to be available for randomization in the study. A stratified randomization procedure was used to assign the selected schools to either active or passive surveillance. Participants who met the criteria for being exposed to TB were referred to the diagnostic ward for pulmonary tuberculosis confirmation. A total number of 3441 males and 3202 females between the ages 11 and less than 18 years were enrolled into the study.

**Results:**

Of the 3102 participants in the active surveillance group, four subjects were diagnosed with definite tuberculosis, four subjects with probable tuberculosis, and 71 subjects had non-tuberculous Mycobacteria (NTM) isolated from their sputum. Of the 3541 participants in the passive surveillance group, four subjects were diagnosed with definite tuberculosis, two subjects with probable tuberculosis, and 48 subjects had non-tuberculosis Mycobacteria isolated from their sputum. The incidence of definite + probable TB was 147.60 / 100,000 person years in the active surveillance group and 87 / 100,000 person years in the passive surveillance group.

**Conclusion:**

The incidence of pulmonary tuberculosis among adolescents in our study is lower than similar studies conducted in South Africa and Eastern Uganda – countries with a higher incidence of tuberculosis and human immunodeficiency virus (HIV) than India. The study data will inform sample design for vaccine efficacy trials among adolescents in India.

## Background

The incidence of tuberculosis (TB) has declined globally from 139 per 100,000 person years in 2005 to 126 per 100,000 person years in 2013. During the same period, the overall incidence in India declined from 209 to 171 per 100,000 person years [1]. Even so, TB accounts for approximately one million deaths every year globally, according to a recent WHO report, of which 0.24 million deaths were in India. India alone accounts for approximately 24 % of all incident TB cases globally [1]. One of the most effective strategies for the control of tuberculosis is mass vaccination with an effective TB vaccine [2–4]. The Bacillus Calmette-Guérin (BCG) vaccine has been recommended at birth in high burden countries based on its efficacy in reducing TB disease in children, although efficacy wanes with time [5, 6]. Given that exposure to tuberculosis increases with age in high burden countries and that there is an increase in the incidence of TB disease in early adulthood, there is a need for the development of a pre-exposure and/or a post-exposure TB vaccine that could be administered to adolescents prior to adulthood.

TB incidence data in vaccine target populations are important for the design of late-phase vaccine clinical trials. Little is known about the incidence of TB in adolescents in India, although it has been reported that the risk of acquiring TB disease after TB infection is higher in adolescents compared to adults [7]. Based on the age-stratified data available, the new smear positive case notification in 2011 of individuals between 15 and 24 years in India was approximately 0.13 million, which was 20.56 % of total smear positive cases among all age groups in India [8].

The reported incidence of TB disease may be affected by how it was detected. It is possible that in active surveillance (periodic home visits), the disease is picked up at an earlier stage by sputum culture (sputum collected through either gastric lavage or induced sputum) even though the participant might not have symptoms suggestive of tuberculosis. In passive surveillance (where participants self-report for a diagnostic evaluation), it is more likely for TB to be diagnosed at a later stage when symptoms suggestive of tuberculosis are present. While there are obvious advantages to active surveillance, costs and manpower are considerably higher. Therefore, the present methodology was used to determine whether less expensive passive surveillance would yield similar estimates of TB incidence compared to active surveillance. The results would have implications in the study design of large field studies that test new TB vaccine efficacy.

### Objective of the present study

The study was designed to estimate the incidence of pulmonary tuberculosis (PTB) disease among adolescents attending school in South India by using two different surveillance methods (active and passive).

## Methods

### Study setting

The study was conducted in the former Palamaner taluk of Chittoor District of Andhra Pradesh in South India approximately 135 km from Bangalore city from February 2007 to July 2010. The study area for this study consisted of 397 population units, of which 384 were rural. The estimated population at the time of the study was 250,000 [9]. The study area was chosen since the incidence of tuberculosis in this area is fairly similar to national rates in India, and the out-migration rate is low allowing for comprehensive follow-up.

### Study design

The study was a prospective observational cohort study with a 2-year follow-up period.

### Sampling

From an initial list of schools registered with the Government (Mandal) Education Office in the region, a random sampling procedure was used to select a subset of schools to enable approximately 8000 subjects to be available for randomization in the study. A stratified randomization procedure was used to assign the selected schools to either active or passive surveillance. Randomization for the public high schools was stratified by size of school, while the remaining schools were stratified by type of school (private high school, private junior college, or public junior college). The schools not selected during the initial random sampling procedure were also randomized using a similar stratified randomization procedure to either active or passive surveillance and were used only if the targeted number of subjects were not enrolled from the selected subset of schools.

### Study participants

All adolescents aged ≥11 to <18 years attending 58 high schools and four junior colleges (grades 7 through 12) in the study area were approached. The total number of eligible adolescents was 12,388. A decision to work in the schools was made to ensure the consistent conduct of this study and to minimize loss to follow-up.

Participants who indicated that they or their families were likely to move from the study area during the proposed 2 years of follow-up or who indicated that they were unable to attend the follow-up session for the tuberculin skin test (TST) reading at baseline were excluded from the study.

### Study procedures

#### Baseline assessment

At baseline, all participants underwent the following procedures.

##### Questionnaires

The parents or guardians were asked about the participant’s clinical history, including BCG immunization status, history of current or past TB, history of close contact of more than 8 h a week with an adult with proven TB, current signs or symptoms of TB, and history of hospitalizations during the last 6 months and/or diagnosis of acute or chronic diseases. The adolescents were asked to review the medical history provided by their parents or guardians and to provide additional information, if any. Socio-demographic details and indicators were also recorded during the interview, including the parent’s / guardian’s occupation, education, and income; the subject’s gender, religion, date of birth, and whether they were a member of a socially deprived community; and the type of walls found in the subject’s home, type of cooking fuel used, and the presence or absence of a household electricity connection.

##### Clinical assessments

The presence or absence of a BCG scar was noted. Height was measured to the closest 0.10 cm using a standard measuring tape with the subject standing against a wall in the Frankfurt plane; weight was recorded in school clothes but without footwear to the nearest 0.10 kg using a calibrated digital weighing scale (Bhaseen Health Product Pvt. Ltd., Jalandhar, India). Weight and height were used to compute Body Mass Index (BMI) and to derive the BMI-for-age z-scores as a measure of nutritional status. BMI for age Z-score was calculated using the Anthroplus software (Version 3.1, WHO, 2010). Based on the BMI for age Z-score, participants were classified as Underweight (BMI – Age Z-score less than or equal to −2), Overweight (BMI – Age Z-score greater than or equal to 1), or Normal (BMI – Age Z-score between −2 to 1; does not include −2 or 1) [10].

##### Tuberculin skin test

Designated trained research nurses administered 0.10 ml of tuberculin containing two TU Purified Protein Derivative (PPD) of RT 23 with Tween 80 as a stabilizer (SPAN Diagnostic Ltd, Surat, India) on the anterior surface of the forearm about two to four inches below the elbow. A designated trained tuberculin reader made the reading visit between 48 and 96 h after the skin test was administered. A wider window of 4 days for tuberculin skin test evaluation was used when the participant was unavailable for TST reading within 72 h. Approximately 88 % of participants were evaluated for the TST within 48 to 72 h; the remaining participants were examined in the 4 day window. The skin test site was inspected in good light and the maximum transverse diameter of the induration was measured in millimetres using a transparent ruler calibrated in millimetres. The measurements were recorded and documented along with the date and time.

##### Blood sample

All participants provided a 29 ml blood sample, which was processed and stored for immunological analyses. Hemoglobin was estimated at the time of blood sampling using the Hemocue AB (HemoCue India, New Delhi, India).

##### Surveillance of participants

A stratified randomisation procedure assigned the schools to either active or passive surveillance. Schools were chosen as the unit for randomisation, rather than individuals, to avoid contamination of follow-up type.

The participants in the active surveillance group were followed-up every 3 months and asked for any signs or symptoms of pulmonary TB and for any recent exposure to a known case of TB. A blood draw was also performed every 6 months. At the 1 year study visit, participants in the active surveillance group had a repeat TST.

The participants in the passive surveillance group were educated about symptoms related to pulmonary TB and asked to present to the diagnostic ward (DGW) if they developed symptoms and/or were exposed to a known case of tuberculosis.

In addition, all primary health care centres and hospitals in the study area, as well as major referral hospitals outside the study area, were visited once a week to determine whether any of the study participants had been diagnosed with PTB in the facility. To facilitate this process, all participants were given a study ID card, which indicated their enrolment into the study and included the study physician’s contact details. The treating physicians at the health centres were requested to contact the study physician if they suspected PTB in the participant.

At the final study visit (Day 720), the baseline procedures were repeated among all participants in both the active and passive surveillance groups.

#### Confirmation of TB

##### TB confirmation

The participant is diagnosed with pulmonary tuberculosis disease if the individual had (1) at least one positive culture result [Isolation of *Mycobacterium tuberculosis* (Mtb) from either one or both sputum samples using the GenoType MTBC test kit (HAIN kit)] or (2) one sputum smear result positive for acid fast bacilli (AFB) and positive polymerase chain reaction (PCR) for *Mycobacterium tuberculosis* or (3) two sputum smear results positive for AFB, or (4) radiological evidence of PTB on a chest x-ray [Posterior-anterior (PA) view] with concurrent diagnosis by at least two radiologists.

If the participants had a recent history of positive TB contact, had symptoms suggestive of pulmonary TB (unexplained cough, unexplained fever, unexplained weight loss, unexplained night sweats for more than 2 weeks, and Haemoptysis), was diagnosed with pulmonary TB by any other physician, or had a positive TST (TST ≥10 mm) during the baseline procedure or any of the follow-up visits, they were referred to the base hospital for further evaluation of PTB. On arrival to the base hospital, the participant’s clinical history was reviewed and a clinical evaluation performed.

A chest x-ray was taken at the hospital and read by one expert radiologist at St. John’s Medical College. All x-rays that were reported as abnormal and as possible PTB by the first radiologist were read independently by two additional radiologists at the same medical college. Sputum was collected on two consecutive days in a sterile container.

At the laboratory, the consistency of the sputum sample (i.e. salivary, mucoid, muco-purulent and blood stained) was noted. The sputum specimens were examined by concentrated AFB smear microscopy and processed for culture. The media used for the culture of Mycobacteria were Lowenstein-Jensen medium and Mycobacteria growth indicator tube (MGIT) medium. If a specimen was culture-positive, the species of the isolate was confirmed using the GenoType Mycobacterium CM kit and the GenoType MTBC kit (HAIN Lifescience, Germany). Participants who visited the DGW after the re-consent process had an additional 3 ml blood drawn to assess immune response by an in vitro antigen stimulation test (QuantiFERON®-TB Gold In-Tube test) to identify *M. tuberculosis* infection. (Cellestis Limited, Australia, http://www.quantiferon.com/IRM/content/default.aspx).

The final diagnosis and test results were communicated to the study participant’s parent or guardian and medical care provider. If the sputum specimen was smear or culture-positive and/or if the chest x-ray was abnormal and suggestive of PTB, the results and participants were also reported to the government tuberculosis control program (Revised National Tuberculosis Control Programme – Directly observed short chemotherapy (RNTCP-DOTS)) for treatment.

### Case definitions

If the participant had one sputum culture positive for Mtb [Isolation of Mtb from either one or both sputum samples using the GenoType MTBC test kit (HAIN kit)]; OR one AFB-positive sputum smear AND one positive PCR for Mtb; OR two AFB-positive sputum smears, they were considered to have a definite case of PTB. If the participant was not diagnosed as having definite TB but had a clinical assessment compatible with pulmonary TB and the chest X-ray was reported to be “abnormal and suggestive of TB” by at least two of the three radiologists, they were considered to have a probable case of PTB.

### Data management and analysis

All data collected were entered into customized data acquisition software. All data forms were checked for missing data and clarity of data prior to data entry. Double data entry was done and disparities reconciled against the data forms. Descriptive statistics were reported using number and percentages. Total person years of follow-up were calculated. Incidence of TB with 95 % confidence interval (CI) in the active and passive surveillance groups was reported. Chi-square test was used to test the association between types of surveillance, whether the participant attended the DGW for TB screening, and re-consent status to other demographic and clinical characteristics of the subjects. P values less than 5 % were considered statistically significant. All analyses were done using Statistical Package for Social Sciences Version 22.0 (SPSS, 22.0, SPSS Inc, Chicago, IL, USA).

## Results

Of the 12,388 eligible adolescents approached, 3441 males and 3202 females were enrolled; response rates were 53 and 47 % respectively. There were no differences in response rates observed between schools or in the response rates of males and females within a school. Among the 6643 subjects enrolled, 3102 (male 1728, female 1374) were followed in active surveillance and 3541 (male 1713, female 1828) were under passive surveillance. Of these adolescents, 59 % (*N* = 1841) were still in the study at the end of 2 years in the active surveillance group while 68 % (*N* = 1185) were still in the study at the end of 2 years in the passive surveillance group. A total of 1257 (male 698, female 559) and 1127 (male 527, female 600) subjects withdrew from the active and passive surveillance groups respectively. Of the 1257 adolescents who withdrew from the active surveillance group, approximately 2/3 i.e. 68.74 % (*N* = 864) withdrew during the re-consenting process, which many participants found difficult to understand. The remaining 31.26 % (*N* = 393) withdrew for other various reasons. The majority of participants left the study area for education at other institutions, marriage, or with their families. A minority of subjects “felt sick (not TB symptoms) and did not want to continue in the study” (*N* = 3), “had examinations and withdrew from the study” (*N* = 2) and “were fasting during Ramzan (Ramadan) month and withdrew from the study” (*N* = 2). All 1127 adolescents in the passive surveillance group who withdrew from the study withdrew at the time of re-consent. There were six deaths reported: four (male 3, female 1) in the active group and two (male 1, female 1) in the passive group. Verbal autopsies were done for these participants; no cause of death was related to tuberculosis.

**Fig. 1:**
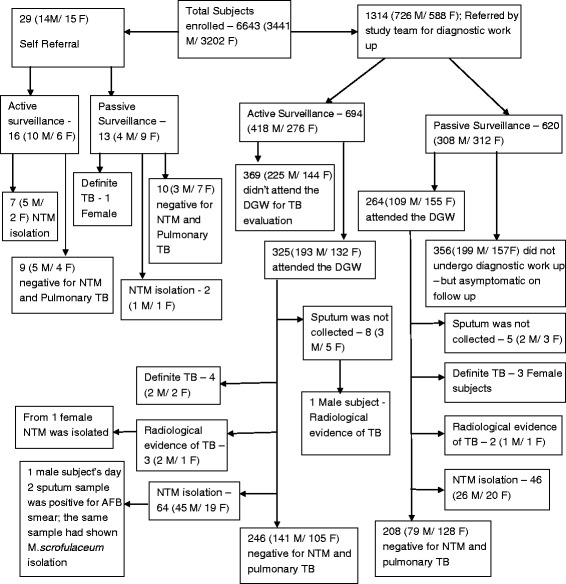
Study profile and TB cases among the adolescent cohort

The baseline characteristics of the study subjects were published earlier [11, 12]. As per the parent’s interview at baseline, out of the 6643 participants, 5674 had BCG immunization at birth and 843 participants had not received immunization. The data of the remaining 126 participants was not available. Figure 1 describes the complete study profile with all TB cases in our adolescent cohort. There were 1314 subjects who were referred to the DGW for the evaluation of pulmonary TB based on the criteria outlined earlier. Of these, 589 subjects attended the diagnostic ward. There were 29 subjects who attended the DGW as self-referrals. Of these 618 subjects screened for PTB, four subjects were diagnosed as having definite TB, four subjects as having probable TB, and 71 subjects had Non-tuberculous Mycobacteria (NTM) isolated from their sputum in the active surveillance group. In the passive surveillance group, four subjects were diagnosed as having definite TB, two subjects with having probable TB, and 48 subjects had NTM isolated from their sputum.

Table 1 describes different characteristics of the 14 definite and probable TB cases. One subject was diagnosed as having definite TB at his 3rd visit to the DGW, while all other subjects were diagnosed at their 1st visit to DGW. Of the eight culture positive cases, five were smear positive.

**Table 1:** Characteristic features of definite and probable TB cases

SI.No	Participant ID	Surveillance	Visit (n)^a^	Gender	Age (years)	BAZ^b^	Smear^c^	Culture^d^	Symptoms^e^				Past TB^e^	Hx TB contact^e, f^	Chest X Ray reult ^g^	QFT Result^h^	TST Value (mm)^i^	Person days of follow up
									Cough	Fever	Haemoptysis	Night sweats						
1	12–104	Active	3	Male	17	Normal	2/2	2/2	+	-	-	-	-	+	3/3	Positive	16	706
2	20–116	Active	1	Female	16	Under weight	2/2	2/2	+	+	-	-	-	-	3/3	NA	15	472
3	33–266	Active	2	Male	15	Normal	2/2	2/2	+	-	-	-	-	-	3/3	Positive	24	718
4	42–107	Passive	1	Female	12	Under weight	2/2	2/2	+	-	+	-	-	-	3/3	NA	7	721
5	72–601	Active	1	Female	15	Under weight	2/2	2/2	+	+	-	+	-	-	3/3	Negative	5	711
6	23–061	Passive	1	Female	17	Normal	0/2	2/2	-	-	-	-	-	-	0/3	Positive	25	725
7	52–301	Passive	1	Female	15	Under weight	0/2	1/2	-	-	-	-	-	-	0/3	Positive	17	725
8	83–013	Passive	1	Female	15	Under weight	0/2	1/2	-	-	-	-	-	-	0/3	NA	23	706
9	37–404	Active	1	Female	15	Normal	0/2	0/2	-	-	-	-	-	-	3/3	Positive	10	706
10	37–430	Active	1	Male	16	Normal	0/2	0/2	-	-	-	-	-	+	3/3	Positive	19	732
11	40–310	Passive	1	Male	16	Under weight	0/2	0/2	-	-	-	-	-	-	3/3	NA	18	706
12	77–503	Active	1	Male	14	Normal	0/2	0/2	-	-	-	-	-	+	3/3	NA	20	729
13	87–155	Passive	1	Female	14	Normal	0/2	0/2	-	-	-	-	-	-	3/3	NA	13	722
14	26–101	Active	1	Male	12	Normal			-	-	-	-	-	-	3/3	Positive	22	710

^a^ The details in the table were corresponding to **n** ^**th**^ visit of the participant

^b^ *BAZ* body mass index for Age Z – score; WHO cut offs 2007, Growth reference 5–19 years; calculated using WHO – ANTHRO software (version 3.2.2); Underweight (<− 2), Overweight (> +1), Normal (−2 to +1)

^c^ 2/2 = two samples smears were positive for Acid fast bacilli of two samples, 1/2 = one sample positive of two, 0/2 = both samples were negative

^d^ 2/2 = two sputum samples had shown the isolation of Mycobacterium *tuberculosis* (M.tb) on Geno Type MTBC test kit (HAIN kit) of two samples; 1/2 = one sample had M.tb of two; 0/2 = both samples were negative for M.tb

^e^ Cumulative response of both parent and subject has been considered in case of history of past TB, TB exposure, Symptoms suggestive of TB

^f^ Recent contact with a TB case at least for 8 h a week; in the period of 3 months

^g^ 3/3 = Radiological evidence of TB - chest x-ray (posterior-anterior view) had been reported as abnormal for TB by three radiologists of three radiologists; 0/3 = No radiologists had reported chest X – ray abnormal for TB

^h^ *QFT* quantiferon, *NA* result Not available

^i^ *TST* tuberculin skin test, the result value corresponds to the nearest one to the diagnostic ward (DGW) visit

The total person years of follow-up in our study was 12,350; 5419 in the active surveillance group and 6931 in the passive surveillance group. The incidence of definite TB, probable TB, and definite + probable TB were 64.80 (95 % CI: 30.10–123.00), 48.60 (95 % CI: 19.70–101.00), and 113.40 (95 % CI: 64.50–185.70) / 100,000 person years respectively in our study. The incidence of definite + probable TB was 147.60 (95 % CI: 68.50–180.30) / 100,000 person years in the active surveillance group and 87 (95 % CI: 35.10–180.00) / 100,000 person years in the passive surveillance group.

Table 2 describes the subjects who attended the DGW for PTB evaluation in both the active and passive surveillance groups. The participants who attended the DGW in the active surveillance group were more likely to be older males with a recent history of weight loss and cough for more than 2 weeks compared to those in the passive surveillance group. The subjects who did not attend the DGW for PTB evaluation even after referral by the study team were more likely to be males, not to have subsequently given their re-consent, and were more likely to be negative for the TST during the follow-up compared to those subjects who attended the DGW for PTB evaluation after referral (Table 3).

**Table 2:** Comparison of active/ passive surveillance group participants to the diagnostic ward in terms of baseline characteristics and other variables

Variable	Active surveillance (*N* = 341) (%)	Passive surveillance (*N* = 277) (%)	*P* value
Gender			
Male	203 (59.5)	113 (40.8)	
Female	138 (40.5)	164 (59.2)	<0.001
Age			
12–13 years	38 (11.1)	33 (11.9)	
14–15 years	171 (50.1)	163 (58.8)	
16–17 years	87 (25.5)	72 (26.0)	<0.001
18–20 years	45 (13.2)	9 (3.2)	
Referral Reason			
Symptoms suggestive of TB	22 (6.5)	4 (1.4)	0.002
Significant TB exposure	17 (5.0)	12 (4.3)	0.84
TST ≥ 10 mm	191 (56.0)	186 (67.1)	0.005
TST conversion	69 (20.2)	54 (19.5)	0.84
Symptoms and TST conversion	1 (0.3)	0 (0.0)	N.A
Contact and TST ≥ 10 mm	2 (0.6)	0 (0.0)	N.A
Symptoms and TST ≥ 10 mm	8 (2.3)	2 (0.7)	0.19
Symptoms and TB contact	2 (0.6)	0 (0.0)	N.A
Self-referral	2 (0.6)	8 (2.9)	0.04
Weight loss ≥ 2 weeks			
Yes	9 (2.6)	1 (0.4)	
No	332 (97.4)	276 (99.6)	0.02
Haemoptysis			
Yes	4 (1.2)	2 (0.7)	
No	337 (98.8)	275 (99.3)	0.69
Cough ≥ 2 weeks			
Yes	38 (11.1)	10 (3.6)	
No	303 (88.9)	267 (96.4)	<0.001
Fever ≥ 2 weeks			
Yes	18 (5.3)	6 (2.2)	
No	323 (94.7)	271 (97.8)	0.05
Night sweats ≥ 2 weeks			
Yes	6 (1.8)	1 (0.4)	
No	335 (98.2)	276 (99.6)	0.13
Subject diagnosed as Pulmonary TB prior to DGW visit			
Yes	1 (0.3)	1 (0.4)	
No	340 (99.7)	276 (99.6)	1.0
Significant TB exposure			
Yes	35 (10.3)	34 (12.3)	
No	306 (89.7)	243 (87.7)	0.44
Caste			
Dalit/ Harijan (socially disadvantaged community)	68 (19.9)	44 (15.9)	
Others	273 (80.1)	233 (84.1)	0.20
Mother’s education			
Illiterate	147 (43.1)	127 (45.8)	
Primary education or more	193 (56.6)	149 (53.8)	0.51
Father’s education			
Illiterate	82 (24.0)	78 (28.2)	
Primary education or more	258 (75.7)	196 (70.8)	0.23
Type of house walls			
Brick	272 (79.8)	235 (84.8)	
Others	69 (20.2)	42 (15.2)	0.11
Type of cooking fuel			
Wood	283 (83.0)	236 (85.2)	
Others	58 (17.0)	41 (14.8)	0.14
Final Diagnosis			
Definite TB	4 (1.2)	4 (1.4)	1.0
Radiological evidence of TB	4 (1.2)	2 (0.7)	0.69
NTM isolated	71 (20.8)	48 (17.3)	0.30
Negative for Pulmonary TB	254 (74.5)	217 (78.3)	0.29

Missing data: Referral Reason–38 (6.1 %), Mother’s education–2 (0.3 %), Father’s education–4 (0.6 %), Final Diagnosis–12 (1.9 %)

*DGW* diagnostic ward, *TST* tuberculin skin test, *N.A* not applicable, Referral reason – Referral criteria to the DGW; Symptoms suggestive of Pulmonary Tuberculosis (PTB) include Cough ≥ 2 weeks, Fever ≥ 2 weeks, Recent Weight loss for ≥ 2 weeks, Night sweats ≥ 2 weeks, Haemoptysis; Significant TB exposure - History of positive TB contact (for more than 8 h/week); Definite TB – Isolation of *Mycobacterium tuberculosis* from the either one or both sputum samples on GenoType MTBC test kit (HAIN kit); Radiological evidence of TB - chest x-ray (posterior-anterior view) had been reported as abnormal for TB by at least two radiologists; NTM (Non-Tuberculous Mycobacteria) isolated from the either one or both sputum samples on GenoType Mycobacterium CM kit

**Table 3:** Comparison of responders/ non responders to the diagnostic ward in terms of baseline characteristics and other variables

Variable	Attended DGW for TB screening^a^ (Responders) (*N* = 589) (%)	Didn’t attend the DGW (Non – Responders) (*N* = 725) (%)	*P* value
Gender			
Male	302 (51.3)	424 (58.5)	0.01
Female	287 (48.7)	301 (41.5)	
Age			
12 years	175 (29.7)	170 (23.4)	0.08
13–14 years	296 (50.3)	391 (53.9)	
15–16 years	95 (16.1)	129 (17.8)	
17–18 years	23 (3.9)	35 (4.8)	
Surveillance			
Active	325 (55.2)	369 (50.9)	0.12
Passive	264 (44.8)	356 (49.1)	
Caste			
Dalit/ Harijan (socially disadvantaged community)	104 (17.7)	139 (19.2)	0.48
Others	485 (82.3)	586 (80.82)	
Mother’s education			
Illiterate	259 (44.0)	347 (47.9)	0.18
Primary education or more	328 (55.7)	378 (52.1)	
Father’s education			
Illiterate	151 (25.6)	214 (29.5)	0.12
Primary education or more	434 (73.7)	506 (69.8)	
Type of house walls			
Brick	482 (81.83)	575 (79.3)	0.25
Others	107 (18.2)	150 (20.7)	
Type of cooking fuel			
Wood	492 (83.5)	629 (86.8)	0.1
Others	97 (16.5)	96 (13.2)	
TST Positivity during 2 years of follow - up			
Yes	545 (92.5)	646 (89.1)	0.03
No	44 (7.5)	79 (10.9)	
Symptoms suggestive of TB during the 2 years follow – up			
Yes	51 (8.7)	54 (7.4)	0.42
No	538 (91.3)	671 (92.6)	
Significant TB exposure during the 2 years follow – up			
Yes	42 (7.1)	35 (4.8)	0.8
No	547 (92.9)	690 (95.2)	
Re - consent Status			
Yes (Given)	546 (92.7)	510 (70.3)	<0.01
No (Not given)	43 (7.3)	211 (29.1)	

Missing data: Mother’s education – 2 (0.2 %), Father’s education – 9 (0.7 %), Re – consent status – 4 (0.4 %)

^a^ Does not include 29 participants who have attended the DGW without referral during the follow–up

*DGW* diagnostic ward, *TST* tuberculin skin test, Symptoms suggestive of Pulmonary Tuberculosis (PTB) include Cough ≥ 2 weeks, Fever ≥ 2 weeks, Recent Weight loss for ≥ 2 weeks, Night sweats ≥ 2 weeks, Haemoptysis; Significant TB exposure - History of positive TB contact (for more than 8 h/week)

Table 4 compares the subjects who re-consented to continue in the study following the change in study sponsors compared to those who withdrew from the study. The adolescents who withdrew were more likely to be 15–18 years age group and in the passive surveillance group. They were also more likely to be negative for TST during the follow-up period, less likely to have symptoms suggestive of PTB, and were less likely to have significant TB exposure during the period under surveillance.

**Table 4:** Comparison of those who re – consented to those who did not re – consent following a change in study sponsorship

Variable	Re – consent given^a^ (*N* = 4647) (%)	Re – consent was not given^b,c^ (*N* = 1937) (%)	*P* value
Gender			
Male	2427 (52.2)	978 (50.5)	0.2
Female	2220 (47.8)	959 (49.5)	
Age			
12 years	1383 (29.8)	392 (20.3)	<0.01
13–14 years	2453 (52.8)	1035 (53.4)	
15–16 years	675 (14.5)	415 (21.4)	
17–18 years	136 (2.9)	95 (4.9)	
Surveillance			
Active	2235 (48.1)	808 (41.7)	<0.01
Passive	2412 (51.9)	1129 (58.3)	
Caste			
Dalit/ Harijan (socially disadvantaged community)	878 (18.9)	340 (17.6)	0.2
Others	3769 (81.1)	1597 (82.4)	
Mother’s education			
Illiterate	2221 (47.8)	954 (49.3)	0.3
Primary education or more	2420 (52.1)	982 (50.7)	
Father’s education			
Illiterate	1210 (26.0)	501 (25.9)	0.86
Primary education or more	3416 (73.5)	1430 (73.8)	
Type of house walls			
Brick	3674 (79.1)	1545 (79.8)	0.52
Others	973 (20.9)	392 (20.2)	
Type of cooking fuel			
Wood	3990 (85.9)	1653 (85.3)	0.58
Others	657 (14.1)	284 (14.7)	
TST Positivity during the follow - up			
Yes	949 (20.4)	240 (12.4)	<0.01
No	3698 (79.6)	1697 (87.6)	
Symptoms suggestive of TB during the follow – up			
Yes	96 (2.1)	7 (0.4)	<0.01
No	4551(97.9)	1930 (99.6)	
Significant TB exposure during the 2 years follow – up			
Yes	65 (1.4)	12 (0.6)	0.01
No	4582 (98.6)	1925 (99.4)	
Referral to DGW Status			
Attended the DGW	546 (11.7)	43 (2.2)	<0.01
Didn’t attend the DGW even after referral	510 (11.0)	211 (10.9)	
Visited DGW even without referral during follow - up	25 (0.5)	4 (0.2)	

Missing data: Mother’s education – 7 (0.1 %), Father’s education – 27 (0.3 %)

^a^ Among the 4647 adolescents one death was reported after re-consent process in active surveillance group which is also included in the table

^b^ Fifty-nine subjects in active surveillance group whose day 720 form filled (closure of the study) before the re – consenting process data was considered as missing data. Of these three deaths were reported

^c^ There were two deaths reported after re-consent process in the passive surveillance group whose number is included in the table

*DGW* diagnostic ward, *TST* – tuberculin skin test, Symptoms suggestive of Pulmonary Tuberculosis (PTB) include Cough ≥ 2 weeks, Fever ≥ 2 weeks, Recent Weight loss for ≥ 2 weeks, Night sweats ≥ 2 weeks, Haemoptysis; Significant TB exposure - History of positive TB contact (for more than 8 h/week)

## Discussion

In our study, the incidence density of definite and probable pulmonary TB among adolescents was 113 per 100,000 person years. The overall incidence of tuberculosis reported by WHO in India for all age groups was 170 per 100,000 person year population in 2012, which is greater than our study results [8]. A lower incidence might be expected in school-going adolescents than in the general population [8]. School-going children may also be healthier than their counterparts who drop-out of school due to socio-economic reasons. Data (2012–13) from the district in which this study was conducted indicate that the enrolment for students between standards I-V was 92 %, VI to VII 84 %, and VIII to X 75 %. The majority of our enrolments in the study were between standards VII and X [13]. School enrolment and drop-out rates vary across ages and gender [14]; being female and reasons related to poverty and economic reasons were major factors associated with drop-outs [15]. Thus, our study sample is likely to have had a smaller proportion of the very poor compared to the general population, which may be also the reason for lower incidence in our study.

In our study, the incidence of culture-positive PTB was 65 per 100,000 person years. Data in an earlier study conducted by the Tuberculosis Research Center, Chennai, Chingleput district, South India was 90 per 100,000 person year population among 10–24 year olds during the period of 1984–1986 [16]. The reduction in incidence may be attributed to a secular change in the incidence with the implementation of Revised National Tuberculosis Control Programme and the smaller age range of our study subjects. Similar studies in adolescents conducted in Eastern Uganda and South Africa show an overall incidence of 235 per 100,000 person years and 450 per 100,000 person years respectively [17, 18]. However, these countries have a higher prevalence of TB in general, compared to India, and higher rates of human immunodeficiency virus (HIV). HIV status was not evaluated in our study population; however, data from the area indicate that the HIV prevalence among antenatal clinic attendees in Chittoor district between 2010 and 2011 was 0.50 to 1.00 % and the prevalence of HIV in Andhra Pradesh state among adults was 0.90 % [19].

Reliable epidemiological data on TB among adolescents is essential for accurate study design and planning for vaccine efficacy trials [20–22]. In India, the only available data among the adolescent age group, until now, is RNTCP data. Our study data aims to supplement the RNTCP data and to provide the additional details needed to conduct TB vaccine trials among adolescents in India.

The strengths of our study are the relatively large sample size, the detailed work up at baseline, the in-depth diagnostic evaluation, and the follow-up of participants in all clinics and hospitals within and surrounding the study area. The data available about response rates of schools at the time of enrolment into the study suggest that there is unlikely to be a responder bias; however, detailed health history was unavailable for the non-responders to exclude this bias completely. The major limitation was the loss to follow-up that followed the change in the study sponsor that led to re-consenting subjects. Approximately 59 % were still in the study at 2 years in the active surveillance group and 68 % remained in the passive surveillance group. However, we do not believe that this loss to follow-up would have led to a decrease in the numbers of TB patients we detected, since weekly surveillance of clinics and hospitals within the study area and in the surrounding areas after the re-consent period did not reveal any new PTB cases from within our project area in the study age group. Another limitation was that only 44.80 % of the patients referred to the diagnostic ward for screening actually underwent the procedures. However, we do not believe that this would have resulted in a significant underestimate of the incidence given that during follow-up in the field, the 55.20 % of participants who did not undergo the diagnostic procedures did not worsen in health and did not attend any clinics or health centres under surveillance during the study. Indeed, the surveillance of the health centres was undertaken to particularly address the likelihood that participants might not be seen by the study physicians but might access other health providers in and around the study area. Fifty four participants who were suspected for TB and had symptoms suggestive of TB at the 2 year follow-up did not attend the diagnostic ward for TB diagnosis confirmation. These participants and others who did not attend the diagnostic ward were considered to not have TB in the analysis.

## Conclusion

The incidence of pulmonary tuberculosis among adolescents in our study is much lower than similar studies conducted in South Africa and Eastern Uganda, which may be partly attributed to low incidence of HIV in India. The study data with higher incidence of PTB in active surveillance compared to passive surveillance method will help inform the design of future TB vaccine efficacy trials among adolescents in India.

## Abbreviations

ARTI, annual risk of tuberculosis infection; AOR, adjusted odds ratio; BCG, Bacillus Calmette Guerin; BMI, body mass index; CI, confidence intervals; DOTS, directly observed short chemotherapy; DGW, diagnostic ward; HIV, human immunodeficiency virus; Mtb, *mycobacterium tuberculosis*; MGIT, mycobacteria growth indicator tube; MTBC, *mycobacterium tuberculosis* complex; NTMs, non-tuberculous mycobacteria; OR, odds ratio; PTB, pulmonary tuberculosis; RNTCP, revised national tuberculosis control programme; TST, tuberculin skin test; TU, tuberculin units; TB, tuberculosis; TBTSG, TB trials study group; WHO, World Health Organization
